# Exercise-induced methylation of the *Serhl2* promoter and implication for lipid metabolism in rat skeletal muscle

**DOI:** 10.1016/j.molmet.2024.102081

**Published:** 2024-12-08

**Authors:** Mutsumi Katayama, Kazuhiro Nomura, Jonathan M. Mudry, Alexander V. Chibalin, Anna Krook, Juleen R. Zierath

**Affiliations:** 1 Department of Physiology and Pharmacology, Karolinska Institutet, Stockholm, Sweden; 2 Division of Diabetes and Endocrinology, Department of Internal Medicine, Kobe University Graduate School of Medicine, Kobe, Japan; 3 Section of Integrative Physiology, Department of Molecular Medicine and Surgery, Karolinska Institutet, Stockholm, Sweden

**Keywords:** Skeletal muscle, Exercise training, DNA methylation, Promoter activity, Serine hydrolase like 2, Lipid metabolism

## Abstract

**Objectives:**

Environmental factors such as physical activity induce epigenetic modifications, with exercise-responsive DNA methylation changes occurring in skeletal muscle. To determine the skeletal muscle DNA methylation signature of endurance swim training, we used whole-genome methylated DNA immunoprecipitation (MeDIP) sequencing.

**Methods:**

We utilized endurance-trained rats, cultured L6 myotubes, and human skeletal muscle cells, employing MeDIP sequencing, gene silencing, and palmitate oxidation assays. Additional methods included promoter luciferase assays, fluorescence microscopy, and RNA/DNA analysis to investigate exercise-induced molecular changes.

**Results:**

Gene set enrichment analysis (GSEA) of differentially methylated promoter regions identified an enrichment of four gene sets, including those linked to lipid metabolic processes, with hypermethylated or hypomethylated promoter regions in skeletal muscle of exercise-trained rats. Bisulfite sequencing confirmed hypomethylation of CpGs in the *Serhl2* (Serine Hydrolase Like 2) transcription start site in exercise-trained rats. *Serhl2* gene expression was upregulated in both exercise-trained rats and an "exercise-in-a-dish" model of L6 myotubes subjected to electrical pulse stimulation (EPS). *Serhl2* promoter activity was regulated by methylation and EPS. A *Nr4a* binding motif in the *Serhl2* promoter, when deleted, reduced promoter activity and sensitivity to methylation in L6 myotubes. Silencing *Serhl2* in L6 myotubes reduced intracellular lipid oxidation and triacylglycerol synthesis in response to EPS.

**Conclusions:**

Exercise-training enhances intracellular lipid metabolism and phenotypic changes in skeletal muscle through epigenomic modifications on *Serhl2*. Hypomethylation of the *Serhl2* promoter influences *Nr4a* transcription factor binding, promoter activity, and gene expression, linking exercise-induced epigenomic regulation of *Serhl2* to lipid oxidation and triacylglycerol synthesis.

## Highlights

• Exercise training hypomethylates *Serhl2* promoter in rat skeletal muscle.
• Exercise training upregulates *Serhl2* mRNA expression in rat skeletal muscle.
• *Serhl2* promoter activity is regulated by methylation via a Nr4a binding motif.
• *Serhl2* regulates intercellular lipid metabolism and lipid droplet formation.

## 1 Introduction

Sedentary lifestyles are rapidly increasing worldwide, placing a heavy burden on healthcare systems [1,2]. Exercise training interventions are first-line treatment against the development of type 2 diabetes and associated complications by enhancing insulin sensitivity and improving body weight management [3]. Skeletal muscle plasticity is affected by exercise at the molecular level through signaling pathways coupled to transcription/translation machinery, which ultimately alters mRNA and abundance of proteins that control substrate metabolism [4]. While numerous exercise-responsive signal transducers have been identified, there remains a knowledge gap in charting and validating the vast array of exercise-induced molecules and features. Deciphering these molecular transducers will provide mechanistic insight into the exercise-responsive networks exerting control over functional and metabolic adaptations.

Evidence is emerging that exercise affects skeletal muscle metabolism and performance through epigenetic modifications [[5], [6], [7], [8], [9]]. Epigenetic modifications, such as cytosine–phosphate–guanine (CpG) methylation, are essential marks controlling cell fate and function [10]. DNA methylation often leads to silencing of genes as exemplified by X-chromosome inactivation and imprinting [11]. DNA hypomethylation has also been described in cells grown in the presence of agents that alter differentiation [12,13]. Non-heritable changes in the epigenome have regulatory consequences by modulating the expression of genes controlling cellular function [14]. Therefore, efforts are underway to characterize the epigenomic landscape and tissue-specific responses to environmental perturbations including physical activity, to elucidate the transcriptional control of growth and metabolism.

Genome-wide approaches provide an unbiased view of the effects of exercise, diet, or metabolic status on DNA methylation in skeletal muscle [[5], [6], [7], [8], [9],[15], [16], [17], [18], [19], [20]]. This open-ended strategy is useful to enable the discovery of environmentally triggered modifications in the epigenome, without being restricted to studies of the canonical pathways and targets typically selected *a priori* for investigation. We have identified time- and intensity-dependent influences of exercise training on DNA methylation and gene transcription in healthy young men and women [5,7]. Nevertheless, the mechanisms by which exercise-associated DNA methylation events regulate transcriptional activity and the skeletal muscle phenotype have not been fully investigated.

Exercise increases lipolysis of both adipose tissue and intramuscular triacylglycerols. Intramyocellular lipids are stored lipid droplets, which are found at central and peripheral regions within muscle fibers, with both morphology and distribution of lipid droplets influencing skeletal muscle insulin sensitivity and lipolysis [21,22]. In skeletal muscle, lipolysis is driven by adipose triglyceride lipase (ATGL), hormone-sensitive lipase (HSL), and monoacylglycerol (MAG) lipase, which sequentially produce diacylglycerol (DAG), MAG and glycerol, respectively [23]. Thus, intramyocellular lipids represent important fuel sources during endurance exercise training. Disentangling the molecular transducers of exercise-induced lipid metabolism through unbiased omics profiling may provide novel insights into skeletal muscle function.

Here we compared the whole-genome DNA methylation landscape of skeletal muscle between sedentary or exercise-trained rats and identified promoter regions of genes that were differentially methylated after exercise training. *Serhl2* (Serine Hydrolase Like 2) promoter methylation was downregulated, with reduced CpG site methylation of the promoter, and increased gene expression in exercise-trained rats. *Serhl2* expression was upregulated in an *exercise-in-a-dish* model of rat L6 myotubes. *Serhl2* mRNA expression and promoter activity were regulated through a *Nr4a* binding motif. Here, we establish the role of this exercise-responsive protein in contraction-induced lipid metabolism and lipid droplet formation and uncover a functional role for *Serhl2* in transducing exercise-training effects in skeletal muscle lipid metabolism.

## 2 Materials and methods

### 2.1 Animals and exercise protocol

The study protocols were approved by the regional animal ethics committee in Stockholm, Sweden. The cohort has been previously studied to elucidate the molecular mechanism by which exercise training increased insulin responsiveness for glucose transport and effects on skeletal muscle Na^+^-K^+^-ATPase subunit expression [24]. Rats were randomly assigned to two subgroups: exercise-trained or sedentary control. Endurance training consisted of two 3-hour swim bouts, separated by a 30-minute rest, for five consecutive days. Rats were anesthetized with an intraperitoneal injection of pentobarbital sodium (5 mg/100 g body weight) 16 h after the last exercise bout. Tissues were collected and frozen in liquid nitrogen and *gastrocnemius* muscle was used for subsequent analysis.

### 2.2 L6 cell culture and differentiation

L6 rat myoblasts were obtained from the American Type Culture Collection and grown in α-Minimal Essential Medium (α-MEM) containing 10% (vol/vol) FBS and 1% Antibiotic-Antimycotic (Thermo Fisher Scientific, Waltman, MA). Cells were plated in 6-well plates. After 24 h (day 0), myoblasts were cultured in differentiation medium (α-MEM, containing 2% horse serum and 1% Antibiotic-Antimycotic) for 5 days before harvesting as myotubes.

### 2.3 Primary human skeletal muscle culture and differentiation

Satellite cells were isolated from *vastus lateralis* skeletal muscle as described (30). Cell cultures were maintained at 37 °C under 7.5% CO_2_ as myoblast cells. Myoblast cells were differentiated into myotubes as described [25].

### 2.4 Electrical pulse stimulations (EPS)

Differentiated myotubes grown in 6-well plates were exposed to EPS (*exercise-in-a-dish*) at 40 V, 2 ms, 1 Hz for 3 h per day, using the C-Pace EP Culture Pacer (IonOptix, Westwood, MA). The EPS exposure was conducted over 1 or 5 days, with non-stimulated cells as control. Cells were collected for experiments immediately after the last EPS exposure.

### 2.5 Genomic DNA and RNA extraction

Genomic DNA of rat *gastrocnemius* muscle was extracted with DNAeasy Blood and Tissue Kit (Qiagen, Hilden, Germany). Total RNA was extracted from rat *gastrocnemius* muscle and cultured myotubes with E.Z.N.A. Total RNA Kit (Omega Bio-tek, Norcross, GA). DNA and RNA concentration were determined with a Nanodrop 1000 (Thermo Fisher Scientific).

### 2.6 Whole genome methylated DNA immunoprecipitation (MeDIP) sequencing

Whole genome methylated DNA immunoprecipitation (MeDIP) sequencing was used as an unbiased approach to identify genes regulated by endurance exercise training in rat *gastrocnemius* muscle. MeDIP-sequencing and initial alignment was performed by Arraystar Inc. (Rockville, MD). A MeDIP-sequencing library preparation was constructed as described [26]. Genomic DNA was sonicated to ∼100-500 bp with a Bioruptor sonicator (Diagenode, Liège, Belgium). Sonicated DNA (300 ng) was end-repaired, A-tailed, and ligated to single-end adapters following the standard Illumina genomic DNA protocol. After agarose size selection to remove unligated adapters, the adaptor-ligated DNA was used for immunoprecipitation using a rat monoclonal anti-5-methylcytosine antibody (Diagenode). DNA was heat-denatured at 94 °C for 10 min, rapidly cooled on ice, and immunoprecipitated with 1 μl primary antibody overnight at 4 °C with rocking agitation in 400 μl immunoprecipitation buffer (0.5% BSA in PBS). To recover the immunoprecipitated DNA fragments, magnetic beads (200 μl) were added and incubated for an additional 2 h at 4 °C with agitation. Thereafter, five washes were performed with ice-cold immunoprecipitation buffer. A nonspecific rat IgG immunoprecipitation was performed in parallel as a negative control. Washed beads were resuspended in Tris–EDTA buffer with 0.25% SDS and 0.25 mg/ml proteinase K for 2 h at 65 °C and then allowed to cool to room temperature. DNA was purified using Qiagen MinElute columns and eluted in 16 μl elution buffer (Qiagen). PCR (14 cycles) was performed on 5 μl of the immunoprecipitated DNA (5 μl) using single-end Illumina PCR primers (Illumina, San Diego, CA). The resulting reactions were purified with Qiagen MinElute columns, after which a final size selection (300–500 bp) was performed by electrophoresis in 2% agarose. Libraries were quality controlled by Agilent 2100 Bioanalyzer. An aliquot of each library was diluted in elution buffer to 5 ng/μl and 1 μl was used in PCR reactions to confirm the enrichment for the methylated region.

### 2.7 DNA sequencing and analysis of sequencing data

The library was denatured with 0.1 M NaOH to generate single-stranded DNA, which was loaded onto channels of a flow cell at 8 pM and amplified *in situ* using TruSeq Rapid SR Cluster Kit (#GD-402-4001, Illumina). Sequencing was carried out by running 36 cycles on Illumina HiSeq 2000 according to the manufacturer's instructions. Thereafter, the stages of image analysis and base calling were performed using Off-Line Basecaller software (OLB V1.8). After passing a Solexa CHASTITY quality filter, the clean reads were aligned to the rat genome (UCSC RN5) using BOWTIE software (V0.12.8). To quantify the DNA methylation level of any specific region, we extended each uniquely aligned read to 250 bp in length. A methylation score (MeDIP-score) for any region in the genome was defined as the number of extended reads per kb as described [27]. The DNA methylation status of a specific region was defined as unmethylated if the MeDIP-score was less than 9.48 reads·kb^−1^, as partially methylated if the MeDIP-score was between 9.48 and 50.62 reads·kb^−1^, and completely methylated if the MeDIP-score was greater than 50.62 reads·kb^−1^. Regions related to a gene were annotated as such: as a promoter region if localized within −700 bp to +200 bp from the transcription start site (TSS); as a gene body region if localized from +2000 bp downstream of the TSS to the transcription termination site (TTS), if the gene was longer than 3k bp in length. CpG islands, defined as at least 200 bp in length and with a G + C content of 50% and a CpG frequency (observed/expected) of 0.6, were analyzed and annotated independently. Sequencing data were submitted to Gene Expression Omnibus (GEO) and the submission ID is GSE250067.

### 2.8 Gene set enrichment analysis (GSEA) and transcription factor motif search

GSEA by Broad Institute, version (4.1.0) was used to link genes identified in a specific gene group with their occurrence in biological pathways or processes. The rank gene list was further annotated using MSigDBv5.0 (http://www.broadinstitute.org/), which contains curated functional gene sets of various biological states [28,29]. Individual matches of transcription factor motifs were scanned using JASPAR core vertebrates [30].

### 2.9 Gene expression analysis

RNA was reverse transcribed using the High-capacity cDNA RT Kit (Thermo Fisher Scientific) and Real-time PCRs were performed in duplicate using SYBR green-based probes in a StepOnePlus detector (Thermo Fisher Scientific). The geometrical mean of *Beta-actin* and 36B4 were used as an endogenous control for rat and human genes, respectively. Custom-made SYBR green qRT-PCR primers were designed for qRT-PCR analysis, synthesized, and are reported in Table S1 and Table S2.

### 2.10 Bisulfite conversion and pyrosequencing

Genomic DNA was bisulfite treated using the EpiTect Fast Bisulfite Conversion Kit (Qiagen). PCR primers were designed using PyroMark primer design software (Qiagen) and PCR was performed using the PyroMark PCR kit (Qiagen). The annealing temperature was optimized for every primer set. PCR products were visualized by electrophoresis on a 2% agarose gel to confirm the identity of the product (size) and to estimate the amplification success (intensity). Pyrosequencing was performed using a PyroMark Q24 system (Qiagen). Non-CpG cytosines were used as internal controls for bisulfite conversion efficiency. Data are reported as percentage methylation by determining the number of times the site existed as cytosine in the context of the total number of times the site was detected as thymine or cytosine. Data were analyzed using PyroMark Q24 software (Qiagen). Primer sets for PCR and pyrosequencing are reported in Table S3.

### 2.11 Promoter luciferase assay in L6 myotubes

The promoter region of the *Serhl2* gene (nucleotides −250 to +120 bp and +1/+120 bp [relative to the transcription start site]), was subcloned into the pGL3-Basic Vector (Promega, Madison, WI) to yield the *Serhl2* promoter-luciferase plasmid. The plasmids in which the Nr4a binding motif was deleted were constructed using site-directed mutagenesis. Mutations were verified by sequencing analysis. L6 myotubes were transfected with a plasmid encoding β-galactosidase as an internal control. Luciferase and β-galactosidase activities in cell lysates were measured 48 h after transfection using the Luminescent β-Galactosidase Detection Kit II (Takara Bio, Kusatsu, Japan). Luciferase activity was normalized by β-galactosidase activity. The assay was performed in duplicate, and results reported as mean ± SEM.

### 2.12 *In vitro* methylation of constructs

Luciferase reporter constructs were produced using *dam*^*−*^*/dcm*^*−*^ bacteria strain SCS110 (Stratagene, La Jolla, CA), which lacks two methylases found in *E. coli*. The constructs for the luciferase assay were methylated *in vitro* by M. *SssI* CpG DNA methylase (New England BioLabs, Beverly, MA) in the presence of 160 μM of S-adenosylmethionine at 37 °C for 3 h. The methylation status of each construct was determined by *Hha* I digestion.

### 2.13 Gene silencing

L6 myotubes were transfected at day 1 and 3 post-induction of differentiation, with 200 nM of either silencer select Negative control No.1, silencer select siRNA s179864 to target *Serhl2*, or silencer select s132917 to target *Nr4a3* (Life Technologies, Foster City, CA). Transfections were performed for 12 h in OptiMEM reduced serum media with Lipofectamine 3000 (Invitrogen, Carlsbad, CA) for *Serhl2* silencing and Lipofectamine RNAiMAX transfection reagent (Invitrogen) for *Nr4a3* silencing. Cells were harvested 2 days after the final transfection and silencing efficiency was determined by qPCR as described above.

### 2.14 Palmitate oxidation and triacylglycerol analysis

Palmitate oxidation was determined in L6 myotubes as described [31]. Cells were incubated with 25 μM non-labeled palmitic acid and [9,10-^3^H] palmitic acid for 6 h. The amount of ^3^H_2_O released into the culture media was determined by scintillation counting. Results were normalized to total cellular protein content (Pierce BCA Protein Assay Kit, Thermo Fisher Scientific) and scintillation counting was determined using a 1414 WinSpectral Liquid Scintillation Counter. Triglyceride composition was determined in L6 myotubes as described [32]. L6 myotubes were incubated with 0.2 μCi/ml [^14^C(U)] palmitate (Perkin Elmer, CA) with non-radioactive palmitate (25 nM) for 6 h and the extracted lipids were separated by thin layer chromatography (TLC) (Silica Gel G 250 μm 20 × 20 cm TLC plates, Analtech, DE). The TLC plates were exposed to an X-ray film and incubated at −80 °C for two weeks. The films were developed and bands corresponding to triglycerides were quantified by densitometry using Quantity One software (Bio-Rad Laboratories Inc., Hercules, CA).

### 2.15 Fluorescent microscopy

Lipid droplets were visualized using Bodipy 493/503 (Thermo Fisher Scientific). Nuclei were stained with DAPI (Sigma–Aldrich, St. Louis, MO). Briefly, L6 myoblasts were rinsed twice with PBS, fixed with 4% formalin for 10 min, incubated in 2 μM Bodipy 493/503 solution in PBS for 15 min, and after washing twice with PBS, incubated in 0.1 mg/ml DAPI. Cells were visualized by fluorescent microscopy (Zeiss Axio Vert A1). Images were captured and analyzed using Image J version 1.53a software (NIH, USA).

### 2.16 Human gene expression data set availability

Datasets of human skeletal muscle (GSE117070 and GSE19420) were extracted from the GEO database of the National Center for Biotechnology Information platform (https://www.ncbi.nlm.nih.gov/geo). The GSE117070 dataset contained transcriptional profiling of *vastus lateralis* skeletal muscle from healthy individuals before and after exercise training [33]. From the GSE19420 dataset derived from human *vastus lateralis* skeletal muscle, we extracted the data from type 2 diabetes patients before and after exercise training [34]. GEO2R was used to compare the differential expression of SERHL2 gene between the pre- and a post-training groups (https://www.ncbi.nlm.nih.gov/geo/ge2r) [35].

### 2.17 Statistical analysis

Data are presented as mean ± SEM. Methylation data was analyzed using two-way ANOVA and other data was analyzed using paired Student's *t*-test. Analyses were performed using GraphPad version 10.0 (GraphPad Software, Inc., La Jolla, CA). Statistical significance was set at *P* < 0.05.

## 3 Results

### 3.1 Whole methylation profiles and identification of DMRs in skeletal muscle

**Figure 1:**
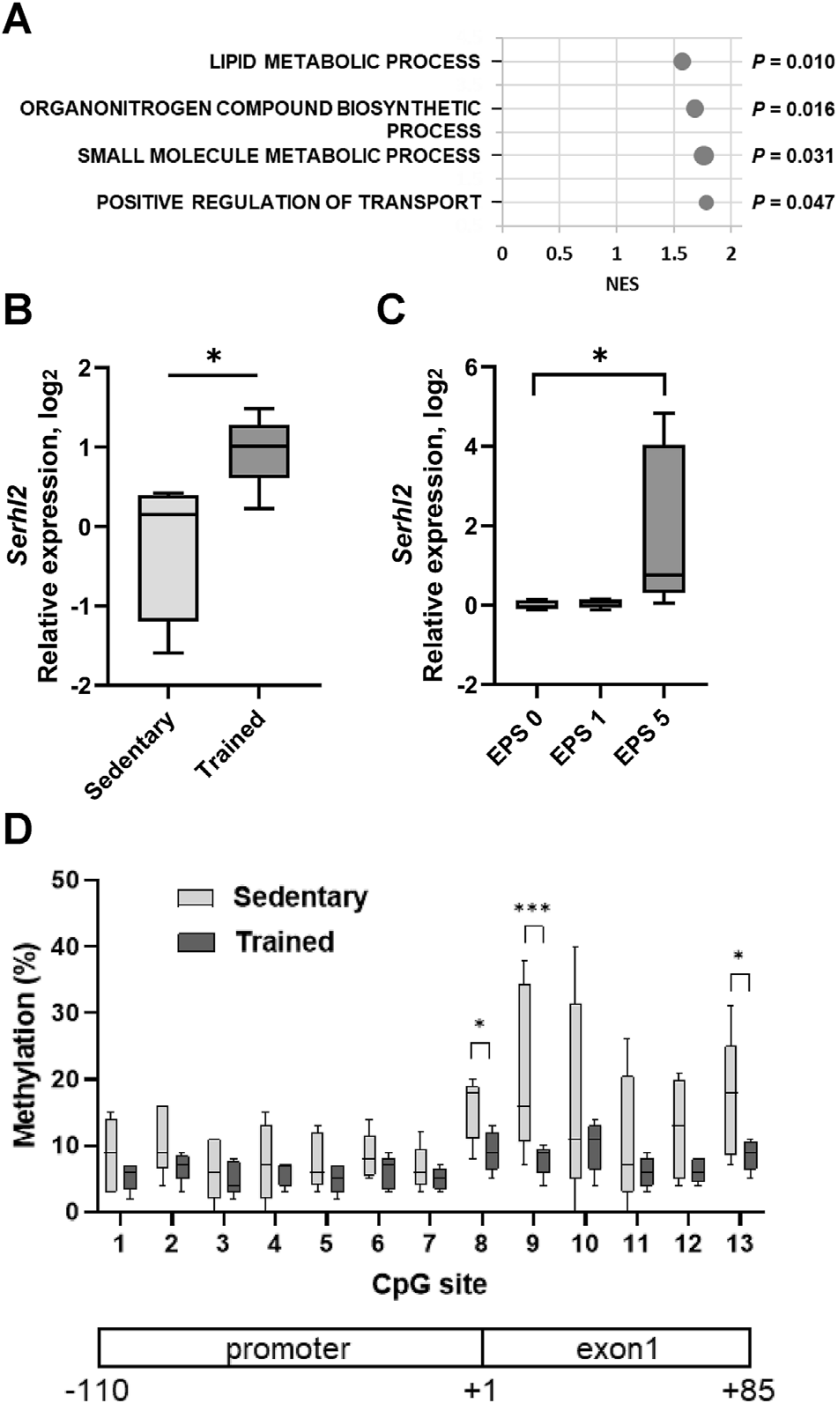
**Exercise effects on metabolic processes from the analysis of differentially methylated regions in sedentary and trained rat skeletal muscle, highlighting exercise-induced promoter hypomethylation and increased gene expression of Serhl2.** (A) Gene set enrichment analysis (GSEA) of 243 promoters of differentially methylated regions (DMRs) in skeletal muscle of sedentary versus exercise-trained rats. (B) *Serhl2* mRNA expression in skeletal muscle from sedentary versus exercise-trained rats. ∗*P* < 0.05. (C) *Serhl2* mRNA expression in an *exercise-in-a-dish* model (myotubes stimulated EPS). EPS was applied to cultured L6 rat myotubes for 3 h per day for 1 (EPS 1) or 5 days (EPS 5), with non-stimulated cells were used as control (EPS 0, control). ∗*P* < 0.05. (D) Bisulfite pyrosequencing DNA methylation of rat skeletal muscle from sedentary versus exercise trained rats at individual CpGs of *Serhl2*. Schemas under graphs showed the structure of gene promoter and first exon, and CpG positions investigated in this report. Sedentary: light gray; Trained: dark gray. Data are mean ± SEM. ∗*P* < 0.05, ∗∗∗*P* < 0.0005 for exercise effect within one single CpG.

To investigate exercise-induced changes in skeletal muscle DNA methylation, we trained rats using a five day swim protocol, previously reported to increase the abundance of proteins involved in glucose metabolism and mitochondrial biogenesis, as well as enhancing glucose transport capacity, insulin-stimulated glycogen storage, and oxidative metabolism [24,36,37]. Whole-genome methylated DNA immunoprecipitation (MeDIP) sequencing was used to determine whether endurance exercise training alters DNA methylation in skeletal muscle. We analyzed DNA methylation status and identified differentially methylated regions (DMRs) linked to annotated genes in skeletal muscle of exercise-trained rats compared to sedentary rats (Supplementary Fig. S1 and Supplementary Table S4, S5 and S6). While MeDIP sequencing analysis detected many methylated regions at baseline (sedentary), differences between sedentary and endurance trained rats were modest, with ratios of DMRs of 1.8% in ‘promoter region’, 3.2% in ‘GpG’ and 0.4% in ‘gene body’ (Fig. S1). Thus, the overall methylation profile was not markedly affected by exercise training in rat skeletal muscle. We next focused our analysis on DMRs in promoter regions and identified 327 gene promoters (*P* < 0.05 and Fold change >1.5) with altered methylation (Supplementary Table S4). Amongst these 327 promoters, we focused on 243 promoters (MeDIP score >10) to build a reliable dataset for our subsequent analysis. These 243 genes were subjected to gene set enrichment analysis (GSEA), which identified four gene sets that were enriched after endurance training, including three gene sets related to lipid metabolic processes and one gene set related to transport regulation process (Figure 1A). Based on this analysis, we prioritized for further validation, twenty genes related to lipid metabolism with differentially hypermethylated promoter regions and seven genes with differentially hypomethylated promoter regions in the −500/+500 promoter region (Supplementary Table S7). We measured mRNA expression of these twenty-seven genes and selected five genes that showed significant and inverse changes between promoter methylation and mRNA gene expression. From these 27 genes, we selected five candidates (four which were hypermethylated *Ahcyl1*, *Dguok*, *Eif4h* and *Smpd4*, and one which was hypomethylated, *Serhl2,* following exercise training) for further validation using pyrosequencing (Supplementary Table S7). Pyrosequencing confirmed the promoter hypomethylation for *Serhl2.* Conversely, the changes in methylation of *Ahcyl1*, *Dguok*, *Eif4h* and *Smpd4* promoters that were identified by MeDIP could not be recapitulated by pyosequencing. Given the inverse relationship between Serhl2 promoter methylation and mRNA expression and previous reports that *Serhl2* is induced by passive stretch of skeletal muscle *in vivo* [38], we prioritized *Serhl2* for further validation of exercise-induced changes in gene expression and promoter methylation.

### 3.2 Exercise alters promoter methylation and gene expression of *Serhl2* in rat skeletal muscle

The methylation status of promoter regions can affect gene expression. We found that *Serhl2* mRNA expression was upregulated in skeletal muscle after endurance training (Figure 1B). To confirm whether muscle contraction directly upregulates *Serhl2* mRNA, we subjected rat L6 myotubes to electrical pulse stimulation (EPS). EPS mimics some of the exercise responses observed in skeletal muscle, such as changes in gene expression and glucose uptake [39,40], and therefore this constitutes an *in vitro exercise-in-a-dish* model system. *Serhl2* mRNA was upregulated after 5 days of EPS (Figure 1C). In addition, we evaluated DNA methylation of CpG sites in *Serhl2* gene promoter regions (9 CpG sites within the proximal *Serhl2* promoter and 4 CpG sites within exon 1 of *Serhl2* gene) and confirmed result of the global methylation using pyrosequencing (Figure 1D). Methylation of the *Serhl2* gene promoter at CpG sites 8, 9, and 13 was significantly reduced in skeletal muscle from endurance trained versus sedentary rats. A trend for reduced methylation of the *Serhl2* gene promoter at CpG site 10 and 12 in skeletal muscle from endurance trained versus sedentary rats was noted, but this did not reach statistical significance. Although EPS of L6 myotubes increased *Serhl2* mRNA, promoter methylation was unaltered (Supplementary Fig. S2), possibly because the genome DNA turnover rate is faster in L6 myotubes versus adult skeletal muscle. Overall, we found that with both exercise training and in our *exercise-in-a-dish* model, *Serhl2* mRNA expression was increased, and in intact rat skeletal muscle, this was concomitant with reduced methylation of the promoter region close to the transcription start site (TSS). These results provide evidence to suggest that muscle contraction drives these epigenomic changes.

### 3.3 Human *SERHL2* expression is upregulated with EPS in human skeletal muscle cells

**Figure 2:**
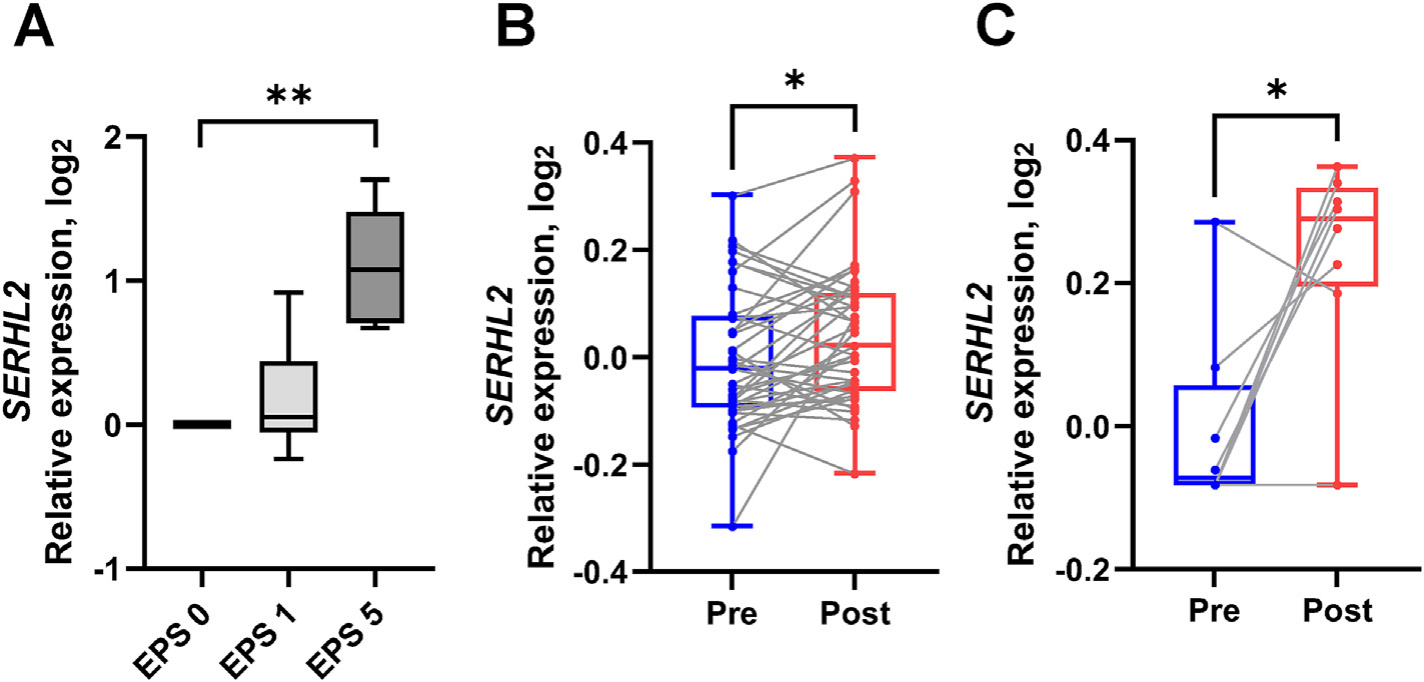
**Effects of effects on *SERHL2* mRNA in human myotubes and skeletal muscle**. (A) *SERHL2* mRNA expression in an *exercise-in-a-dish* model of human myotubes. (B) *SERHL2* mRNA expression in human skeletal muscle from healthy individuals after twenty weeks of endurance training (GSE117070). Pre-training (blue). Post-Training (red). (C) *SERHL2* mRNA expression in human skeletal muscle from people with type 2 diabetes (T2DM) after 52 weeks of endurance training (GSE19420). Pre-training (blue). Post-Training (red). ∗*P* < 0.05; ∗∗*P* < 0.005.

*Serhl2* gene was originally cloned in mouse as *Serhl*, with rat *Serhl2* and human *SERHL2* identified based on gene homology [38]. Since gene and protein homology are highly conserved between species, with similar gene structures in each respective genome, we tested whether EPS or exercise training alters *SERHL2* expression in human myotubes and human skeletal muscle. In EPS-exposed primary human myotubes, SERHL2 expression was increased at Day 5 (Figure 2A). Next, we accessed publicly available data sets from the Gene Expression Omnibus (GEO) to interrogate the exercise training response of human skeletal muscle. While acute exercise or short-term exercise training did not alter SERHL2 expression, SERHL2 expression was increased in skeletal muscle from healthy individuals following a fully supervised long-term progressive 20-week endurance training program (GSE117070, Figure 2B) [33]. Furthermore, SERHL2 expression was increased in skeletal muscle of people with type 2 diabetes following a 52-week extensive exercise training intervention (GSE19420, Figure 2C) [34]. Thus, *SERHL2* mRNA levels remain unchanged following acute or short-term exercise training but increase in response to prolonged endurance exercise training.

### 3.4 Skeletal muscle contraction activates the *Serhl2* promoter

**Figure 3:**
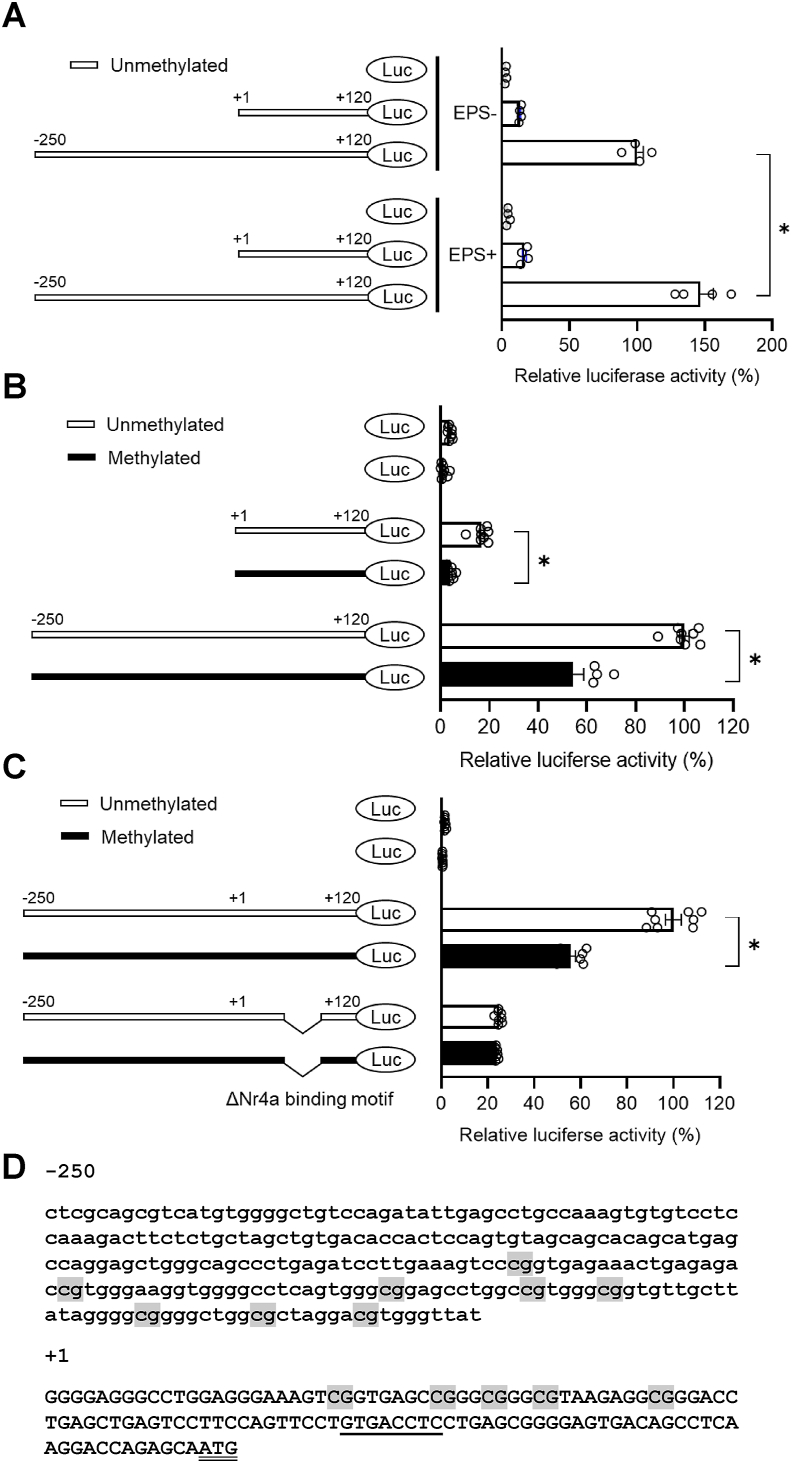
**Schematic representation of *Serhl2* promoter-luciferase constructs and promoter activity in L6 myotubes**. Schematic overview of *Serhl2* promoter fragments subcloned upstream to a luciferase reporter gene. Numbers refer to the nucleotide of the promoter region of the *Serhl2* relative to the transcription start site. (A) *Serhl2* promoter activity in L6 myotubes transfected with each promoter construct. Cells were subjected to EPS one day before performing the luciferase assay (EPS+). Control cells were not subjected to EPS (EPS-). Methylated (■) or unmethylated (□) sequences of the *Serhl2* promoter region were used as a promoter region in the promoter assay. (B) Effects of methylation on *Serhl2* promoter activities. Methylated (■) or unmethylated (□) sequences of the *Serhl2* promoter region were used as a promoter region in the promoter assay. (C) Effects of deleting an Nr4a binding motif and methylation on *Serhl2* promoter activity. Methylated (■) or unmethylated (□) sequences of the *Serhl2* promoter region were used as a promoter region in the promoter assay. (D) Schematic representation of CpG sites and *Nr4a* binding motif in the *Serhl2* promoter region (nucleotides −250 to +120 bp, relative to the transcription start site. The CpG sites investigated by the pyrosequencing analysis are highlighted (gray), the Nr4a binding motif is underlined, and the ATG start codon is double underlined. Data are presented as the mean ± SEM. ∗*P* < 0.05.

Since exercise resulted in increased *Serhl2* mRNA and hypomethylation of the promoter region in rat skeletal muscle, we investigated whether muscle contraction and methylation directly regulated the promoter activity. Rat skeletal muscle L6 myoblasts were transfected with a luciferase promoter plasmid containing the upstream region of *Serhl2* (Figure 3). Our analysis revealed that the −250/+120 construct displayed substantial promoter activity, which was further increased in response to EPS (Figure 3A). To determine the promoter regions directly involved in the induction of *Serhl2* gene transcription, we next studied a +1/+120 deletion construct. The +1/+120 deletion construct retained some luciferase activity, although not as high as the complete −250/+120 construct. While the activity of the −250/+120 construct was further increased by EPS, the +1/+120 deletion construct did not show any EPS-dependent change in transcriptional activity. Thus a 120-bp region neighboring to the TSS of *Serhl2* is necessary for promoter activity, and a 250-bp upstream region from the TSS is responsible for the promoter activity that is responsive to EPS.

### 3.5 *In vitro* methylation of the *Serhl2* promoter abolishes promoter activity in L6 myoblasts

Since endurance training resulted in hypomethylation of the *Serhl2* promoter region and increased mRNA in rat skeletal muscle, we investigated whether methylation directly regulated *Serhl2* promoter activity. A methylated *Serhl2* promoter-luciferase construct was transfected into L6 myoblasts (Figure 3B). Methylation exerted an inhibitory effect on promoter activity on both the −250/+120 *Serhl2* and the +1/+120 *Serhl2* construct. The inhibitory effect of methylation on the +1/+120 *Serhl2* construct was larger than on the −250/+120 *Serhl2* construct. These results provide evidence that sequences in the +1/+120 *Serhl2* construct are essential for promoter activity, with methylation directly attenuating promoter activity.

### 3.6 Nr4a binding motif near the TSS of *Serhl2* regulates promoter activity

To determine whether there are specific transcription factors binding motifs in the *Serhl2* promoter, we performed an analysis using JASPAR, which returned twenty putative transcription factor motifs for thirteen transcription factors (Supplementary Table S8). Among these, *Nr4a2* was suggested to have two binding sites in the *Serhl2* promoter region with high confidence. *Nr4a2* is a member of the nuclear receptor 4A (Nr4a) subfamily: *Nr4a1*, *Nr4a2* and *Nr4a3*. These three transcription factors are highly homologous in the DNA binding domain (around 95%). Thus, these nuclear receptors may bind to a similar or common DNA motif in regulating genes [41]. *Nr4a1–3* mRNA levels are rapidly increased in skeletal muscles after acute exercise [42]. We identified Nr4a protein binding motifs in the *Serhl2* promoter region, with the putative binding motif located close to a TSS and 28 base pairs from CpG site 13 (Figure 3D). We constructed a deletion mutant of the Nr4a binding motif from the −250/+120 *Serhl2* luciferase construct and determined the effects of methylation on promoter activity. While methylation reduced activity of the −250/+120 *Serhl2* luciferase construct, deletion of the Nr4a binding motif led to a greater decrease in promoter activity both in the absence and presence of methylation (Figure 3C). Our results suggest that Nr4a regulates *Serhl2* mRNA by binding to its promoter.

### 3.7 Exercise training increases *Nr4a3* mRNA

**Figure 4:**
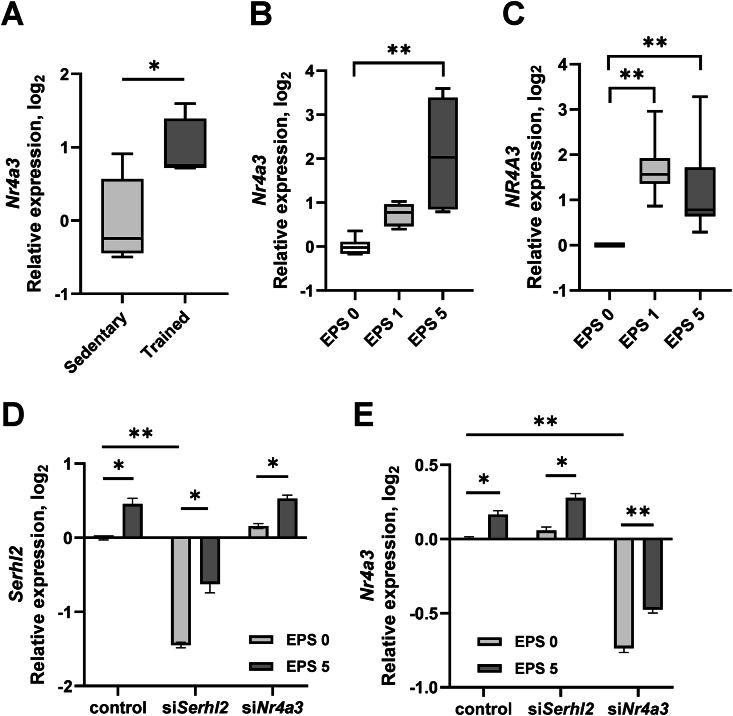
**Relative gene expression of *Nr4a3*/*NR4A3* and *Serhl2***. *Nr4a3* mRNA expression in skeletal muscle of (A) sedentary or exercise-trained rats or (B) L6 myotubes treated with EPS. (C) *NR4A3* mRNA expression in human myotubes treated with EPS. Myotubes (B and C) were exposed to EPS for 1 or 5 days. (D) *Serhl2* mRNA expression in si*Serhl2*-silenced or si*Nr4a3*-silenced L6 myotubes. (E) *Nr4a3* mRNA expression in si*Serhl2-*silenced or si*Nr4a3*-silenced L6 myotubes. Data are mean ± SEM. ∗*P* < 0.05, ∗∗*P* < 0.005.

We have previously identified *NR4A3* as one of the most exercise- and inactivity-responsive genes [43]. Given that *Nr4a3* regulated *Serhl2* promoter activity in an *exercise-in-a-dish* model of L6 myotubes, we determined the effects of exercise on *Nr4a3* mRNA. *Nr4a3* expression was increased in rat skeletal muscle after endurance exercise training (Figure 4A) and following EPS in both L6 myotubes (Figure 4B) and primary human myotubes (Figure 4C).

### 3.8 *Nr4a3* is dispensable for the EPS-mediated upregulation of *Serhl2* mRNA

To determine whether *Nr4a3* controls *Serhl2* expression through the Nr4a binding motif in the *Serhl2* promoter we next studied the mechanism of gene regulation by silencing *Nr4a3* in L6 myotubes. EPS increased *Serhl2* mRNA even in *Serhl2* silenced L6 myotubes (Figure 4D), likely because *Nr4a3* mRNA was also increased by EPS in the *Serhl2* silenced L6 myotubes (Figure 4E). However, EPS also increased *Serhl2* mRNA in *Nr4a3* silenced L6 myotubes (Figure 4D). Collectively, these results provide evidence to suggest that *Serhl2* transcription is controlled jointly by the Serhl2 promoter methylation and the presence of the Nr4a binding region, rather than a change in *Nr4a3* abundance.

### 3.9 *Serhl2* regulates EPS-induced lipid metabolism

**Figure 5:**
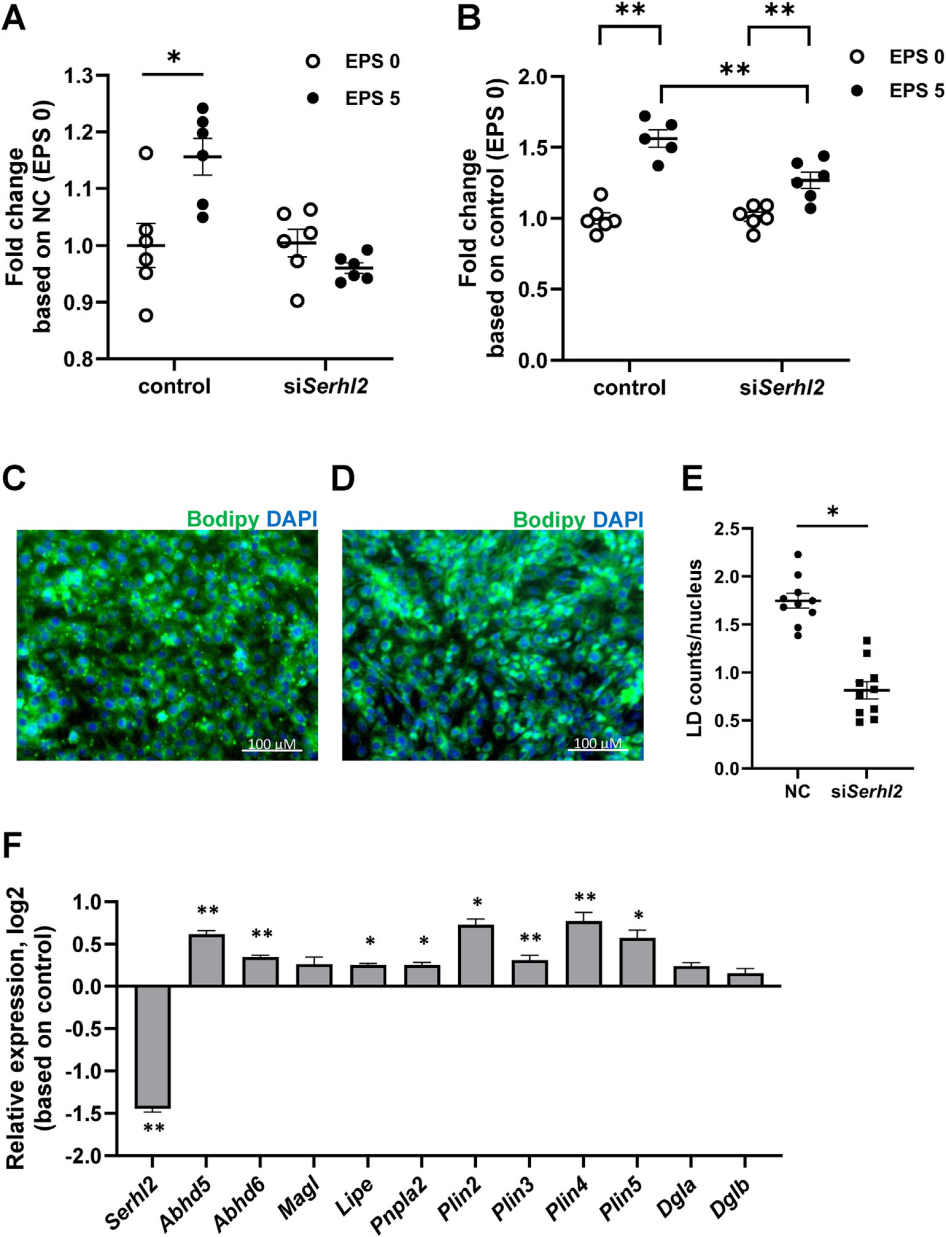
**Effects of *Serhl2* silencing on lipid metabolism in L6 myotubes or myoblasts**. Fatty acid oxidation (A) and fatty acid incorporation into triacylglycerol (TAG) (B) in *Serhl2*-silenced L6 myotubes. Microscopic images show representative views in which lipid droplets (LDs) stained with Bodipy 493/503 (green) and 4′,6-diamidino-2-phenylindole (DAPI) (blue) respectively in control cells transfected with a scrambled sequence (C) or in *Serhl2*-silenced L6 myoblasts (D). The number of LDs in control or *Serhl2*-silenced cells were quantified (E). mRNA expression of *Serhl2* and genes involved in lipid metabolism was determined in control or *Serhl2*-silenced L6 myotubes (F). Data are mean ± SEM. ∗*P* < 0.05, ∗∗*P* < 0.005.

The amino acids structure of Serhl2 is highly homologous with serine hydrolases, including Abhd6 or Magl2, enzymes known to both hydrolyze lipids and control lipid metabolism [44,45]. Thus, we determined the effects of *Serhl2* silencing on fatty oxidation in L6 myotubes (Figure 5A). While EPS increased palmitate oxidation in L6 myotubes, *Serhl2* silencing reduced this effect. In *Serhl2* silenced L6 myotubes, the EPS-induced increase in TAG content was reduced (Figure 5B). Collectively, our results provide evidence to suggest that *Serhl2* plays a role in exercise-induced lipid metabolism.

### 3.10 *Serhl2* reduced lipid droplet number and expression of lipid metabolism-related genes

*Serhl2* silencing reduced fatty acid oxidation and TAG content, concomitant with morphological changes of lipid droplets (LDs). Given that LD's serve as metabolic hubs, which contain intramuscular TAG in skeletal muscle, we next stained cells with Bodipy 493/503 to label cellular neutral lipids. *Serhl2* silencing reduced the number of LDs labeled with Bodipy 493/503 (green particles) (Figure 5C,D). The number of Bodipy 493/503 labeled LDs was decreased when compared against the total number of LDs (Figure 5E). This suggested *Serhl2* regulates LD formation and functions. Next, we measured the expression of genes implicated in lipid droplet function and serine hydrolases homologous to *Serhl2* including *Abhd5* (α/β-Hydrolase Domain-containing protein 5), *Abhd6* (α/β-Hydrolase domain-containing protein 6) and *Magl* (Monoacylglycerol Lipase) (Figure 5F). Abhd6 and Magl are monoglyceride lipases, Abhd5 is a lipase coactivator and both of these enzymes play a role in lipid metabolism [[44], [45], [46]]. While *Abhd5* and *Abhd6* mRNA was increased in *Serhl2* silenced L6 myotubes, *Magl* mRNA was unaltered. Additionally, we measured the expression of several genes involved in lipolysis including *Lipe* (Lipase E also known as hormone-sensitive lipase (*Hsl*)), which catalyze the hydrolysis of TAGs, diacylglycerols (DAGs), monoacylglycerols (MAGs), and *Pnpla2* (Patatin like phosphatase domain containing 2, also known as adipose triglyceride lipase (ATGL)), which catalyzes TAGs over DAGs [47]. *Lipe* and *Pnpla2* mRNA was increased in *Serhl2* silenced L6 myotubes. Expression of *Plin*s (Perilipins) family members, which are proteins located on the surface of lipid droplets with key roles in the formation and the maintenance of the LDs, were also increased. Conversely, expression of other lipases that catalyze DAGs over MAGs, including diacylglycerol lipase alpha and beta (*Dgla* and *Dglb*), were unaffected. Thus, we establish a role for *Serhl2* in lipid droplet formation and metabolism in skeletal muscle, with implications for the biochemistry of exercise.

## 4 Discussion

To provide greater insight into the molecular transducers of the exercise training response we performed a genome wide MeDIP-sequencing analysis of skeletal muscle from endurance trained rats and determined the effects on DNA methylation and transcription. In this model, skeletal muscle methylation was modestly affected by endurance exercise training, corroborating previous methylome analysis of human skeletal muscle [48]. Notably, the degree of exercise-induced methylation varies considerably between studies [5,9,49] raising the possibility that the type of exercise modality, the duration of the training intervention, the previous conditioning of the individual, or other factors related to the training response may influence any effect of exercise training on the epigenome.

Our analysis of promoters enriched in DMRs identified genes related to cellular lipid metabolism pathways that were affected by exercise training. Skeletal muscle is one of the main regulators of lipid metabolism. Triglycerides, stored in lipid droplets in skeletal muscle are hydrolyzed to free fatty acids, thereby providing a fuel for working muscles during exercise [50]. Specifically, we found that exercise training altered promoter methylation of a previously uncharacterized gene, namely *Serhl2* (Serine Hydrolase Like 2). *Serhl2*, was identified in murine skeletal muscle as a member of the serine hydrolase family, induced by passive stretch [38]. However, its function has been largely unknown until now. Thus, exercise training modifies methylation of promoters of lipid metabolism genes in skeletal muscle.

We found that the promoter region of *Serhl2* was hypomethylated and mRNA expression was increased after exercise training as well as EPS in cultured myocytes. Thus, our results indicate that muscle contraction directly regulates epigenomic methylation event in the myocyte, rather than other cell types. Based on time-course data of repeated EPS over 5 days, as well as the exercise training studies in humans, changes in *Serhl2* mRNA, and presumably methylation, represent an adaptive response to training, rather than an acute response. Conversely, acute exercise leads to altered mRNA and methylation of genes associated with mitochondrial function and glucose metabolism including PGC1α, Tfam, and PDK4 in skeletal muscle of young men and women, as well as isolated mouse skeletal muscle and primary human myotubes exposed to contraction [5]. Different modes of acute exercise and exercise training have distinct time courses and transcriptional fingerprints [43,51]. Thus, future studies should focus on elucidating the time course, epigenomic changes, and molecular mechanisms of regulation in skeletal muscle.

Using a luciferase construct we establish that *Serhl2* promoter activity is directly controlled by methylation, with specific regulatory CpG sites identified in skeletal muscle providing molecular insight into exercise-induced regulation of this gene. Moreover, regulatory CpG sites identified by pyrosequencing are in close proximity to an essential Nr4a binding motif on the *Serhl2* promoter. The nuclear receptor 4A subfamily includes *Nr4a1*, *Nr4a2* and *Nr4a3*. These three transcription factors are highly homologous in the DNA binding domain (∼95%) and may bind to a similar or common DNA motif in regulating genes [41]. *NR4A1–3* mRNA levels are rapidly increased in skeletal muscles after acute exercise [42]. *NR4A3* is one of the most exercise- and inactivity-responsive genes in human skeletal muscle [43], however, changes in *Nr4a3* mRNA were without effect on *Serhl2* mRNA. While the molecular transducers of exercise on DNA promoter methylation have yet to be described, our results provide evidence that methylation directly regulates gene expression of exercise-training responsive genes such as *Serhl2*.

Our unbiased analysis identifies novel exercise-training responsive genes. Based on the protein sequence structure, *Serhl2* has been assigned to the α/β hydrolase domain (ABHD) fold super family and proposed to work as a intracellular lipase [52]. While the function and substrates of *Serhl2* are unknown, it shares a high degree of protein homology with ABHD6 which functions as a lipase. The substrate profile resembles Magl, the major 2-arachidonoyl glycerol (2-AG) hydrolase, which plays a role in glucose-stimulated insulin secretion, obesity prevention, and development of metabolic syndromes [[53], [54], [55], [56]]. While *Serhl2* has been proposed to work as a monoacylglycerol lipase, we did not find direct evidence of lipase activity in skeletal muscle. Rather, *Serhl2* gene silencing inhibited EPS-induced fatty acid oxidation and decreased EPS-induced increase of triacylglyceride content in L6 cells. Furthermore, *Serhl2* silencing decreased the number of LDs in L6 cells, concomitant with upregulation of the expression of *Serhl2* homologous genes like Abhd5 and Abhd6 and genes relating to functions of LDs like Lipin family genes. Similar compensatory function of other lipases like HSL have been reported in ATGL knock-out mice [57,58]. Thus, these *Serhl2*-dependent changes in fatty acid oxidation and LD formation reflect the plasticity of skeletal muscle adaptation to inactivity and endurance training [4,59].

## 5 Conclusion

We determined the effect of exercise-training on DNA methylation in rat skeletal muscle and provide evidence that exercise training altered mRNA expression and promoter methylation of a previously uncharacterized gene, namely *Serhl2* (Serine Hydrolase Like 2). Additionally, in an *exercise-in-a-dish* model of cultured L6 myotubes, as well as in people with either normal glucose tolerance or type 2 diabetes, skeletal muscle *SERHL2* mRNA was increased with long time EPS or endurance training, respectively. Thus, exercise-induced changes in *Serhl2*/*SERHL2* expression may be a consequence of long-term training rather than an acute response to exercise. Exercise training increases the number of lipid droplets, reduces individual lipid droplet size located in skeletal muscle, and causes lipid droplet remodeling [60]. Functionally, we provide evidence that *Serhl2* governs lipid metabolism and lipid droplet formation. In cultured L6 myotubes subjected to EPS, we report that the exercise-induced lipid droplet formation occurs following changes in *Serhl2* mRNA expression, which parallels DNA methylation. *Serhl2* promoter activity is directly regulated by methylation and presence of a Nr4a transcription factor binding motif. In conclusion, we validate the functional role of *Serhl2*, a newly described exercise-responsive protein that plays a role in lipid droplet formation. Future studies would assess more aspects of those findings and explain detailed mechanisms by which Serhl2 could function. Our data establishes a link between DNA methylation, transcription, and cellular responses causing morphological changes of intracellular organelles and lipid metabolism.

## Funding

This study was supported by the Swedish Research Council (2015-00165, 2022-00609), Novo Nordisk Foundation (NNF17OC0030088; NNF22OC0077741), Swedish Research Council for Sport Science (P2023-0093), the European Research Council (ERC-2023-AdG 1011420930), a Wallenberg Scholar grant from Knut and Alice Wallenberg Foundation (2023.0312), and the Strategic Diabetes Research Programme at Karolinska Institutet (2009-1068).

## CRediT authorship contribution statement

**Mutsumi Katayama:** Writing – review & editing, Writing – original draft, Methodology, Investigation, Formal analysis, Data curation, Conceptualization. **Kazuhiro Nomura:** Writing – review & editing, Methodology, Investigation, Formal analysis. **Jonathan M. Mudry:** Writing – review & editing, Investigation, Formal analysis. **Alexander V. Chibalin:** Writing – review & editing, Methodology, Investigation, Formal analysis. **Anna Krook:** Writing – review & editing, Writing – original draft, Supervision, Project administration, Funding acquisition, Conceptualization. **Juleen R. Zierath:** Writing – review & editing, Writing – original draft, Supervision, Project administration, Funding acquisition, Conceptualization.

## Declaration of competing interest

The authors declare that they have no known competing financial interests or personal relationships that could have appeared to influence the work reported in this paper.

## Data Availability

Data will be made available on request.

## Appendix A Supplementary data

Multimedia component 1 **Appendix A. (Word file)**. Supplementary data: Figure S1, S2 and Table S1, S2, S3, S7, S8. Multimedia component 1

Multimedia component 2 **Appendix B1. (Excel file)**. Supplementary Table S4. List of the promoters in DMRs. Multimedia component 2

Multimedia component 3 **Appendix B2. (Excel file)**. Supplementary Table S5. List of the CGI in DMR. Multimedia component 3

Multimedia component 4 **Appendix B3. (Excel file)**. Supplementary Table S6. List of the GeneBody in DMR. Multimedia component 4
