# Supplementary material for: Exercise-induced methylation of the Serhl2 promoter and implication for lipid metabolism in rat skeletal muscle
Source: Mol Metab. 2024 Dec 8;92:102081. doi: 10.1016/j.molmet.2024.102081 (PMC11732562; doi:10.1016/j.molmet.2024.102081)
Supplement: Multimedia component 1 — Appendix A. (Word file). Supplementary data: Figure S1, S2 and Table S1, S2, S3, S7, S8. [file mmc1.docx]

**Appendix A. Supplementary data**

**Supplementary Figure S1**

Flowchart for identifying and classifying differentially methylated regions (DMRs) in rat skeletal muscle: comparing sedentary and trained rats. The process included determining DMRs and categorizing them based on genomic location: promoter regions, CpG islands, or gene body regions.


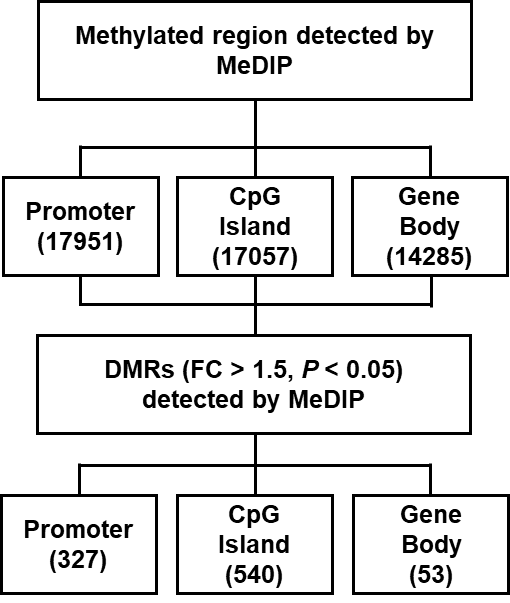


**Supplementary Figure S2**

***Serhl2* promoter methylation profile before and after EPS in L6 myotubes**


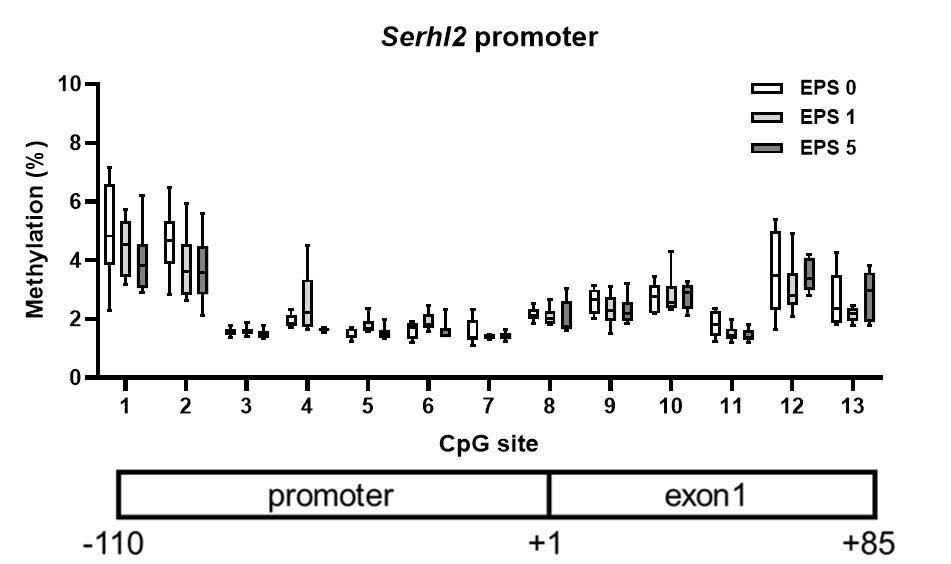


**Supplementary Table S1**. Primers used for real-time qPCR analysis of rat skeletal muscle

| **Target gene** | **Forward primer sequence** | **Forward primer sequence** |
| --- | --- | --- |
| *Abhd5* | GGGCAGCATTGACTCCCTTT | AAAGCTGTCTCACCACTTGGG |
| *Abhd6* | AGCGTCTGCTCCCATCCCCA | TGGCTTGCCAGTGGCGTGAA |
| *Actb* | AGCGTGGCTACAGCTTCACC | AAGTCTAGGGCAACATAGCACAGC |
| *Angpt1* | GCGAGTGCTGGCAGTACAATG | CCGTCGTGTTCTGGAAGAATG |
| *Ahcyl1* | CACGGCTACAACACAGGCTTTG | GGTTCGTGTGGACTGGTCATTG |
| *App* | GATCTCCAACCGTGGCATC | CGTCGACAGGCTCAACTTC |
| *Calm1* | AATCCGTGAGGCATTCCGAG | TAGTTGACCTGTCCGTCTCCATC |
| *Cept1* | TGGATCAACCATAGCAGGAACAAG | TCATGTGTGCAACCACAAGC |
| *Chkb* | CCGTCATTATCTGGCGGAGG | GGCATACCGGCTGATCTCTATC |
| *Cmk* | CTCTGGTCTTCTGGAGTTCCG | AGCATTGGAAGTTGGGGTAGG |
| *Dgla* | TTACAACCTGCGGCATCGG | GGCCAGCAATGATGTCGGA |
| *Dglb* | AGACCCGGGTGCAATGCTGC | GCCCTGGTGTGTGGGTCACG |
| *Dguok* | ACATGGCTTCATCTACCTCCAG | CCTCGAAGTGGAGCTTGGTAG |
| *Dpagt1* | CTGCTCCTGCAGGTTCTGAG | GTCCTGGCTGAGGTCCAATG |
| *Dph1* | CCGACTGGCCAATCAGATCC | TGTAAGGCCACCTTCTTGGC |
| *Egr1* | GAGCACCTGACCACAGAGTC | GGAGAAGCGGCCAGTATAGG |
| *Eif4h* | GTGCGGCTAGTCAGAGACAAAG | AGCCACCCATTCCTCTGTCATC |
| *Fgfr1* | TCTCCGCAGACAGGTAACAG | AAGGCCGAACCAGGAGAAC |
| *Glut4* | GGCTAACATCAGGGTTGGTG | GACAGAAGGGCAACAGAAGC |
| *IL6* | CTCTGGTCTTCTGGAGTTCCG | AGCATTGGAAGTTGGGGTAGG |
| *Lipe* | CCGATTCGCCATAGACCCAG | GTTGGCCAGAGACGATAGCA |
| *Magl* | GCCAATCCGGAATCTGCATC | TGTACAGGTCAACCTCCGAC |
| *Mrpl11* | ACTTCTCCGAGGCGGACTAAG | GATAGAGACACCTCGCTGACC |
| *Mrpl38* | CAGGAGCTTCGTGCCAATTC | GCCGTAGTACTCAGCCAGAC |
| *Myh2* | CTGCTGAAGCAGAGGCAAGT | CCTGAGGTTGGTCATCAGCTT |
| *Myh4* | AGAGTGGCTGAGCAAGAGC | ATCATGGCGGCGTCAGT |
| *Myh7b* | CTCGCATGCAGGACCTAGTG | CTGGGCCTTGCGGTACTTAG |
| *Myod* | ACTACAGCGGCGACTCAGAC | GTGGAGATGCGCTCCACTAT |
| *Myog* | ACGATGGACGTAAGGGAGT | CTACAGGCCTTGCTCAGCTC |
| *Myf5* | GGAATGCAATCCGCTACATT | CAGGGCAGTAGATGCTGTCA |
| *Nr4a3* | ACTGCCCGGTAGACAAGAGA | ACAGATCGGAGGAGATGGTG |
| *Ndufab1* | ACTGACAAGCAAGAGTGAACCAC | AATAGGCCAGATGTGTGAGGAG |
| *Olr865* | GGGTGATTCCAGGGCAGAAC | CATGATGATGAGCCCGTTGC |
| *Olr1119* | GGCGTATGACCGCTATTTGG | CAGCAATACACAGGAACAAGGAC |
| *Pdk4* | TTCCGTCCAGCTGGTGAAG | CCAGCGTGTCTACAAAGTCTGAT |
| *Pgap2* | AGCACACAGATCGCAAGTCC | GGTGTACACTCCAGCCTCAC |
| *Plaa* | GAGCCAGGTACTAGAGAAGGAC | GCACCAGATGAGCCAACAAC |
| *Plin2* | AGCTCCACTCCACTGTCCA | AGTATGTGACTCGATGTGCTCAG |
| *Plin3* | GAAGGTTTCCTGGCCTCGAC | CCACCACACTAGGCTGCAT |
| *Plin4* | CATCTGCCTGGGTGATCTGG | AATCTGCCACCTTGCATCCC |
| *Plin5* | CAGAAGTGGGCTCAGTGGAG | TCTGCATATGCTGGATCAGCTC |
| *Pnpla2* | GCCTGTGTGGAACCGAAAGA | CAGCCACTCCAACAAACGGA |
| *Rwdd3* | AGACAGAAGCAACATCAAGGAGT | TGACTTCAAACGCCAGAAACC |
| *Sacm1l* | AGCATCTGAAGCTGCATATTACAC | GGTGACCTCTGTAGACACGC |
| *Serhl2* | GTGAAGCCTGCTCTCCAGTG | CCAAGATGGAGCCCCCAG |
| *Smpd4* | TGAAGCGGCACATCTCTCATC | GTGTAGAACCTCCAACTTGGCATG |
| *St8sia1* | TGTCATGCGGTGTAACCTTCC | TCACCAACTGAGTCTTGGAACC |
| *Txnrd1* | ACTCGAGGACACAGTTAAGCATG | CAGTTCAGCGAGCCGATATG |
| *Zc3h10* | CGGCGGCAGAACCCAG | CCCAGCTCAGGGTCTTGTTTC |

**Supplementary Table S2**. Primers used for real-time qPCR analysis of human skeletal muscle

| **Target gene** | **Forward primer sequence** | **Forward primer sequence** |
| --- | --- | --- |
| *36B4* | CAGGTGTTCGACAATGGCAG | GGCCAGGACTCGTTTGTACC |
| *NR4A3* | GCCCAGTAGACAAGAGACGT | GAGAGGGCTGAGAAGGTTCC |
| *SERHL2* | AGCTGCTGCAGAGGTTACTG | CACAGCTCCCTGCTGATGAA |

**Supplementary Table S3**. PCR primers used for pyrosequencing

| **Gene name** |  | **Primer sequence** |
| --- | --- | --- |
| *Ahcyl1* | FWD  REV  Seq. | GATTATTGATAAGATAAAGGGGGTGG  ACCCTCATCCCCTAAACA  GATAAAGGGGGTGGT |
| *Dguok* | FWD  REV  Seq. | AGAATAGAGTAGTTTTTGAGGTTTTAT  TACAACCATCCTACCCAAACTTA  GTTAAAATTAGTTATTTTTTGTTTG |
| *Eif4h* | FWD  REV  Seq. | GGGGTAAGGATAGTAAGAGGTAAT  CCCCCAATATAACTCCTCCTCCCC  GTAAGGATAGTAAGAGGTAATT |
| *Serhl2* | FWD  REV  Seq. | GTTGGGTAGTTTTGAGATTTTTGAA  ACTCTAATCCTTAAAACTATCACTC  GTAGTTTTGAGATTTTTGAAAG |
| *Smpd4* | FWD  REV  Seq. | GGTTGGTTTTGAATTTAAGAGATTTATT  TTACTCCCCCCACTCACCTACTTTACTA  TTTAGGTTATTTTTGTGTTATTGA |

**Supplementary Table S7.**  The list of twenty-seven genes selected for validation and subsequent results

| Gene name | MeDIP seq | | qRT-PCR |  | Pyrosequencing | |
| --- | --- | --- | --- | --- | --- | --- |
|  | Fold Change | *P*-value | Fold Change | *P*-value |  |  |
| *Ahcyl1* | 1.9 | 0.0156 | 0.74 | 0.019 | | Not significant |
| *Angpt1* | 1.96 | 0.0411 | 1.49 | 0.21 | | - |
| *App* | 1.77 | 0.0416 | 0.77 | 0.078 | | - |
| *Calm1* | 1.59 | 0.0377 | 0.72 | 0.29 | | - |
| *Cept1* | 1.52 | 0.0185 | 0.98 | 0.62 | | - |
| *Chkb* | 1.87 | 0.0011 | 0.94 | 0.55 | | - |
| *Dguok* | 1.86 | 0.028 | 0.65 | 0.054 | | Not significant |
| *Dpagt1* | 1.87 | 0.0231 | 0.78 | 0.28 | | - |
| *Dph1* | 1.67 | 0.0211 | 1.17 | 0.60 | | - |
| *Eif4h* | 1.98 | 0.0207 | 0.61 | 0.066 | | Not significant |
| *Fgfr1* | 2.40 | 0.0167 | 1.35 | 0.37 | | - |
| *Mrpl11* | 1.96 | 0.002 | 0.86 | 0.36 | | - |
| *Mrpl38* | 1.66 | 0.0107 | 0.91 | 0.48 | | - |
| *Ndufab1* | 2.69 | 0.0142 | 1.07 | 0.96 | | - |
| *Sacm1l* | 1.88 | 0.0009 | 0.85 | 0.13 | | - |
| *Smpd4* | 1.78 | 0.0317 | 0.37 | 0.063 | | Not significant |
| *St8sia1* | 1.69 | 0.0146 | 1.46 | 0.12 | | - |
| *Pgap2* | 1.55 | 0.0343 | 1.26 | 0.40 | | - |
| *Plaa* | 2.39 | 0.0447 | 0.97 | 0.72 | | - |
| *Txnrd1* | 1.78 | 0.0066 | 0.63 | 0.13 | | - |
| *Mthfd2* | 0.55 | 0.021 | 0.90 | 0.48 | | - |
| *Myh7b* | 0.63 | 0.019 | 0.96 | 0.66 | | - |
| *Olr119* | 0.44 | 0.032 | 1.52 | 0.19 | | - |
| *Olr865* | 0.58 | 0.031 | 1.44 | 0.99 | | - |
| *Serhl2* | 0.51 | 0.01 | 2.02 | 0.039 | | Significant hypomethylation |
| *Rwdd3* | 0.65 | 0.027 | 0.72 | 0.34 | | - |
| *Zc3h10* | 0.65 | 0.029 | 1.45 | 0.41 | | - |

**Supplementary Table S8**

Results of transcription factor motif search analysis

| **Matrix ID** | **Name** | **Score** | **Relative score** |
| --- | --- | --- | --- |
| MA0079.2 | SP1 | 12.02 | 0.93 |
| MA0160.1 | NR4A2 | 10.45 | 0.96 |
| MA0105.1 | NFKB1 | 10.40 | 0.89 |
| MA0079.2 | SP1 | 10.29 | 0.89 |
| MA0105.1 | NFKB1 | 9.94 | 0.88 |
| MA0040.1 | Foxq1 | 9.05 | 0.83 |
| MA0156.1 | FEV | 8.91 | 0.89 |
| MA0079.2 | SP1 | 8.70 | 0.85 |
| MA0160.1 | NR4A2 | 8.55 | 0.89 |
| MA0079.2 | SP1 | 8.40 | 0.84 |
| MA0079.2 | SP1 | 8.08 | 0.83 |
| MA0156.1 | FEV | 8.01 | 0.86 |
| MA0018.2 | CREB1 | 7.80 | 0.86 |
| MA0018.2 | CREB1 | 7.35 | 0.85 |
| MA0079.2 | SP1 | 7.34 | 0.82 |
| MA0079.2 | SP1 | 7.33 | 0.82 |
| MA0019.1 | Ddit3::Cebpa | 7.23 | 0.80 |
| MA0102.1 | Cebpa | 7.00 | 0.83 |
| MA0442.1 | SOX10 | 6.81 | 0.91 |
| MA0102.1 | Cebpa | 6.80 | 0.82 |
| MA0079.2 | SP1 | 6.79 | 0.80 |
| MA0099.2 | FOS::JUN | 6.66 | 0.85 |
| MA0117.1 | Mafb | 6.63 | 0.88 |
| MA0099.2 | FOS::JUN | 6.57 | 0.85 |
| MA0102.2 | CEBPA | 6.20 | 0.82 |
| MA0038.1 | Gfi1 | 6.06 | 0.80 |
| MA0152.1 | NFATC2 | 5.96 | 0.80 |
| MA0102.2 | CEBPA | 5.83 | 0.81 |
| MA0117.1 | Mafb | 5.77 | 0.84 |
| MA0117.1 | Mafb | 5.76 | 0.84 |
| MA0442.1 | SOX10 | 5.56 | 0.85 |
| MA0442.1 | SOX10 | 5.56 | 0.85 |
| MA0442.1 | SOX10 | 5.28 | 0.84 |
| MA0117.1 | Mafb | 5.19 | 0.81 |
| MA0117.1 | Mafb | 5.19 | 0.81 |
| MA0117.1 | Mafb | 5.01 | 0.81 |
